# Decoding Deep Learning applications for diagnosis and treatment planning

**DOI:** 10.1590/2177-6709.27.5.e22spe5

**Published:** 2023-01-06

**Authors:** Jean-Marc RETROUVEY, Richard Scott CONLEY

**Affiliations:** 1 University of Missouri - Kansas City, Department of Orthodontics (Kansas City/MO, USA).

**Keywords:** Deep learning, Artificial intelligence, Orthodontics

## Abstract

**Introduction:**

Artificial Intelligence (AI), Machine Learning and Deep Learning are playing an increasingly significant role in the medical field in the 21^st^ century. These recent technologies are based on the concept of creating machines that have the potential to function as a human brain. It necessitates the gathering of large quantity of data to be processed. Once processed with AI machines, these data have the potential to streamline and improve the capabilities of the medical field in diagnosis and treatment planning, as well as in the prediction and recognition of diseases. These concepts are new to Orthodontics and are currently limited to image processing and pattern recognition.

**Objective:**

This article exposes and describes the different methods by which orthodontics may benefit from a more widespread adoption of these technologies.

## RESUMO

**Introdução:**

Inteligência Artificial (AI, de *Artificial Intelligence*), *Machine Learning* (Aprendizado de máquinas) e *Deep Learning* (Aprendizado Profundo) possuem um papel significativo e crescente na área médica do século 21. Essas tecnologias recentes são baseadas no conceito de criar máquinas com potencial de funcionar como um cérebro humano. Isso demanda que uma grande quantidade de dados seja reunida para ser processada. Uma vez processados em máquinas com AI, esses dados têm o potencial de agilizar e potencializar as capacidades de diagnóstico e planejamento do tratamento nas áreas médicas, assim como no diagnóstico e prognóstico de doenças. Esses são conceitos novos na Ortodontia, que atualmente são subutilizados, limitando-se ao processamento de imagens e reconhecimento de padrões.

**Objetivo:**

O presente artigo expõe e descreve os diferentes métodos pelos quais os ortodontistas podem se beneficiar com o uso mais abrangente dessas tecnologias.

## INTRODUCTION

Two major trends are influencing the 21^st^ Century from business and life perspectives. Society has entered a data-driven world where the interactions between machine and humans are intertwined. The importance of artificial and augmented intelligence in our daily lives is becoming increasingly prevalent.^1^ Orthodontics, the most technologically-oriented dental specialty is rapidly adopting these technologies for diagnosis, treatment planning and management of complex malocclusions.^2^ ^-^ ^4^

The development of super computers followed by hypercomputers in the 1990s has allowed large quantity of data to be processed at an extremely rapid rate.^5^ The Artificial Intelligence (AI) field has consequently evolved into machine learning (ML) and deep learning (DL), where computers learn tasks that human are unable to accomplish. 

The field of Orthodontics, with its reliance on 3D data and based heavily on diagnostics and interpretation of large quantity of data from different sources, is particularly well suited to the use of AI and DL.^6^ ^,^ ^7^

## WHAT IS ARTIFICIAL INTELLIGENCE? THE “EARLY DAYS”

**Figure 1:**
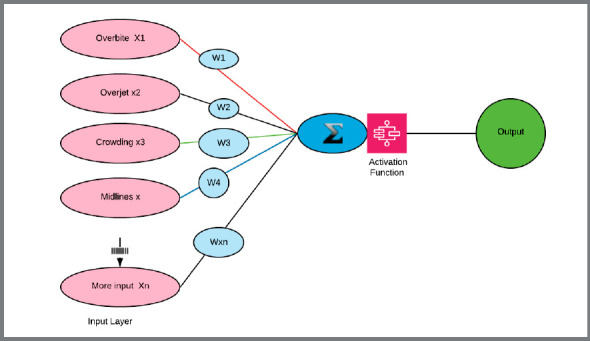
Perceptron, or “artificial neuron”. From left to right: The input layer is used to import the data into the system. The weight (W) are values from 0 to 1 attributed to the input. The Sum Σ is given by the addition of the input multiplied by their respective weights. An activation function is then used to obtain the output.

Dr. Rosenblatt introduced the Perceptron in 1953 and the term “artificial intelligence” first appeared at a conference in 1956.^8^ AI is a large field of computer science where machines learn and interact with human using logical processes based on data.^9^ The concept was to create computers that mimicked human thought process. The Perceptron, or “artificial neuron“, is a linear binary classifier (e.g., yes-no) and is considered the origin of AI (Fig 1).

## ARCHITECTURE OF THE MULTILAYER PERCEPTRON AND OF A SIMPLE NEURAL NETWORK^10^

1. Input initial dataset labeled: Each datapoint is entered, such as overbite, overjet, crowding in Orthodontics.
2. Artificial neuron or unit: Is created to mimic a human neuron and consists of a mathematical function (y = ax +b, as an example), ‘x’ being the inputs, ‘y’ being the output.
3. Layer: The layer consists of a group of artificial neurons placed in a vertical pattern of artificial neurons. Each vertical line of neuron is driven by a specific function.
4. Weights and biases define the strength or importance of the connection of one input to the next input layer. They are adjustable either by the trainer or by multiple iterations in the cycle of learning.
5. Activation function: Mathematical function that helps the network learn by calculating the relative influence of a given artificial neuron on the next layer. These functions are made possible by applying different mathematical algorithms called activation functions, transforming the sum of inputs into outputs.
6. Input layer is the first layer of a neural network. It brings the data gathered into the network, which will be assigned a weight and moved to the next layers. The data can be labelled (organized) or unlabeled (not organized).
7. Hidden layers: The layers that are located between the input layer and the output layer. There may be one or many hidden layers, and each layer acts independently from its predecessor or successor, as they use a different activation function.^11^
8. Output layer represents the final layer. Probable output is the prediction given by the output layer and expressed in terms that are understandable to the user.

Several definitions and subsections of AI have been described: Machine learning, deep learning and reinforcement learning are common terms that are oftentimes used interchangeably, but represent different types of AI.^12^

Machine learning is based on the concept of narrow AI, which is a form of AI programmed by human to perform a specialized task.^13^ ^,^ ^14^ An example is the pursuit of a specific medical diagnosis, where the machine is trained to recognize and classify different signs and symptoms to obtain a probable diagnosis.^15^ ^,^ ^16^ Machine learning can be used for several important functions in Orthodontics: descriptive, predictive, and prescriptive.^17^ ^,^ ^18^ Descriptive functions in Orthodontics can be linked to the formulation of a diagnosis when the different parameters (inputs) are considered^19^. Predictive, as its name implies, is meant to predict the most accurate outcome of a potential treatment - in Orthodontics, it could be the decision to extract or not extract.^20^ Prescriptive functions could be linked to the choice of the most appropriate appliance or biomechanical system to address a given malocclusion. 

Another subset of AI is deep learning, which uses several types of neural networks that learn using unlabeled dataset, without being directly supervised by humans.^21^ ^,^ ^22^ Deep neural networks consist of multiple hidden layers that imitate the cognitive and reasoning abilities of the human brain, to eventually provide general intelligence, which is the closest to human intelligence.^23^ ^,^ ^24^ These networks became relevant in the early 2000s with the advent of more advanced Graphic Processing Units (Nvidia™, 1999) and the creation of very large datasets (Big Data).^9^ ^,^ ^25^ ^,^ ^26^

## ARCHITECTURE OF A NEURAL NETWORK (FIG 2)

 How do neural networks “learn”? 

Why is it important for the clinician?

The premise and promise of any neural network are to analyze data (inputs) through activation functions and multiple neural layers, to produce an output, which can be data classification, regression or prediction, depending on the activation functions applied.^27^ ^-^ ^30^

To create an efficient neural network or model, one must start with data mining and data management.^6^ ^,^ ^31^ The data gathering process involves multiple steps, and is a lengthy procedure.^32^ The first step requires obtaining consistent high-quality data, through correct and optimized methods. The second step is to process, categorize, and classify the data. The third step is to “clean” and structure the data, to optimize it and to avoid issues with future processing.^33^ Once this process is complete, the “clean/optimized” data is now ready to be used for AI processing. Unfortunately, in Orthodontics, there is no centralized data repository or data analysis software readily available, which makes the creation and adoption of deep learning algorithms difficult to implement.^34^ ^,^ ^35^

Orthodontics is particularly well suited for data mining, as it uses multiple related datasets, such as extra and intraoral examination, muscle function, dental casts analysis, cephalometric radiographs, and growth predictions. These separate datasets are difficult to organize and label for the orthodontist, and may be more easily processed with machine learning.^36^ These processes hold the promise of better individualizing diagnosis and treatment planning in the future.^37^ Currently, the profession uses this concept mainly for automated cephalometric analysis and simple treatment predictions. Aligner™ companies use AI to improve processes of tooth alignment and get a better and more predictable outcome. 

There are two basic methods to train a neural network: supervised and unsupervised learning. 

Supervised learning^38^

Supervised learning involves teaching an AI network a single task, such as an object recognition. The data is divided into discrete inputs (e.g., overbite, overjet, number of teeth, crowding, individual cephalometric points or measures) and fed into the neural network. With supervised learning, a segment of the dataset is used to create rules or activation functions, and applied to train the AI network until a satisfactory output is reached. Ninety percent accuracy is the typical minimum threshold. Once the programmed rules and activation functions prove satisfactory, a second dataset called the testing dataset is used to verify the initial network’s accuracy and consistency. Once satisfactory results are achieved with the training data set, the neural network is ready for prediction of future data.^39^

### Unsupervised learning and reinforcement learning

**Figure 2:**
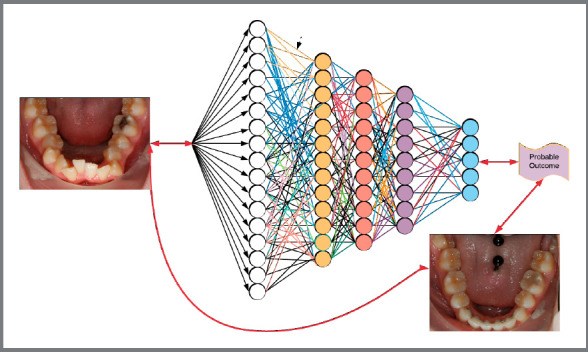
Deep learning neural network.

Unsupervised learning is based on the principles of back propagation and gradient descent (error analysis)^25^ ^,^ ^40^ ^-^ ^42^, in which a large dataset of before and after treatment “inputs” is fed into the neural networks, without specific rules or instructions. Using multiple epochs (epoch is a full circle path of the network layers), the computer determines the correct weights or strengths of the connection(s) to be attributed to each input. The networks independently find correlations and develop the appropriate rules to obtain the most probable output, in relation to the inputs provided. Massive quantities of preferably clean data must be used to find significant correlations not apparent to the human operator. Unsupervised learning utilizes Jacobi’s mathematical principle called “starting with the end in mind”, or “*man muss immer umkehren*”.^43^ Jacobi found that reversing mathematical problems frequently led to their solution, while more conventional approaches led to an impasse. System errors can be limited through careful planning and the use of gradient descent, a function used to find the smallest error in the system^44^ (Fig 2).

## “PREMEDITATION MALLORUM” OR PREMEDITATION OF EVILS (STOICS)^45^

This principle states that one must:^46^

1. Imagine all the negative outcomes that may happen during treatment, and plan for the unexpected.
2. Adopt a worst-case scenario and avoid the “we will figure it out when we get there” approach to any intervention.
3. Anticipate failure for any given treatment approach. A predictive approach rather than a reactive approach is adopted.

## CURRENT USES OF AI BY ORTHODONTIC CORPORATIONS

**Figure 3:**
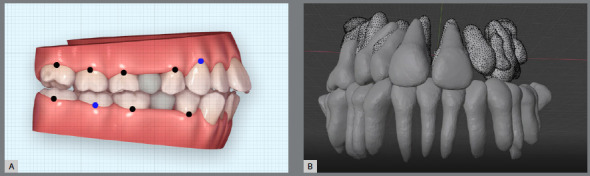
A) Invisalign ClinCheck. B) Blender derived tooth movement from CBCT.

Prior to the introduction of supercomputers, gathering and optimizing large data sets was a complicated task to perform. Invisalign^™^, with its ClinCheck^®^ program, was the first company to introduce easy to visualize outcomes, with a bidirectional (forward and backward) method to simultaneously examine the pretreatment and potential post-treatment outcome (simulation). Superimposition of pre and post models was made available for visualization (Fig 3).^47^ ^,^ ^48^ These private corporations do not share their codes and the software architecture remains a closed one. Recently, open-source software such as Blender (Blender Foundation, Amsterdam, Netherlands) have been adapted to create add-ons to allow the clinicians to create their own simulation and treatment plans.

These add-ons based on the latest and constantly improving open-source technologies hold a lot of promise if the profession invests time and effort in their development.^49^

## AI OR AUGMENTED INTELLIGENCE APPLICATIONS IN ORTHODONTICS

**Figure 4:**
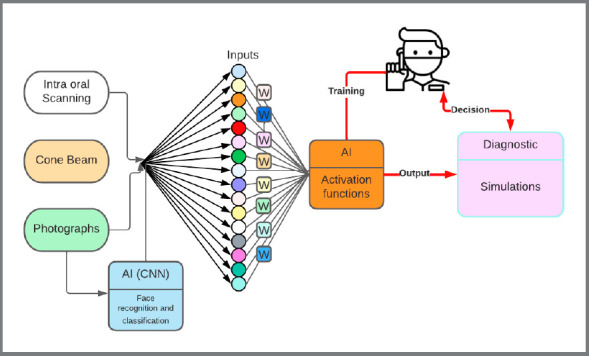
Diagnostic workflow to assist orthodontist in decision making.

Augmented intelligence is a subsection of AI in which the human factor is still predominant, and it uses the analytical and statistical powers of AI to improve outcome.^50^ Presently, supervised learning is used to virtually correct the malocclusion by presenting a probable outcome. With that outcome in mind, we can move backwards to the initial occlusion, in order to predict/foresee and avoid potential hindrances.^51^ The weights are adjusted for each data input to reduce “cost or errors” to predict the true outcome associated to the expected outcome. The orthodontist then decides whether the probabilities of success are high enough before proceeding with the treatment (Fig 4).

The overarching principle is to finish the needed orthodontic correction in a cyber environment, then revert to original condition, to find the most appropriate treatment sequence that will be acceptable to the practitioner. This software opens the door for multiple simulations to be executed by the algorithms using several hundred datapoints. The orthodontist can then review the simulations, make the desired changes, and truly visualize the objectives of treatment. Orthodontics can move from a reactive to proactive treatment management.^52^

## EVOLUTION OF ORTHODONTICS DIAGNOSIS IN ORTHODONTICS 3.0: THE AGE OF DIGITIZATION AND 3D IMAGING

**Figure 5:**
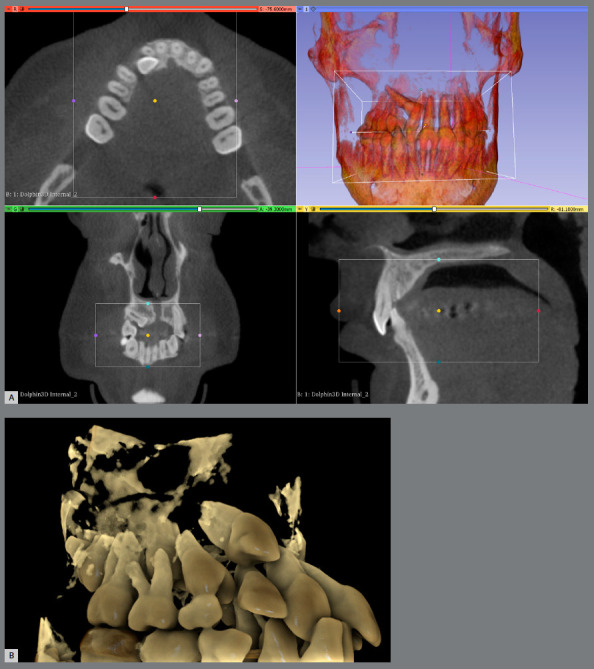
A) CBCT with multiplanar view and 3D rendering. B) Enhanced CBCT image pre-segmentation, using the Drishti software ( The Australian National University, Canberra, Australia ).

**Figure 6:**
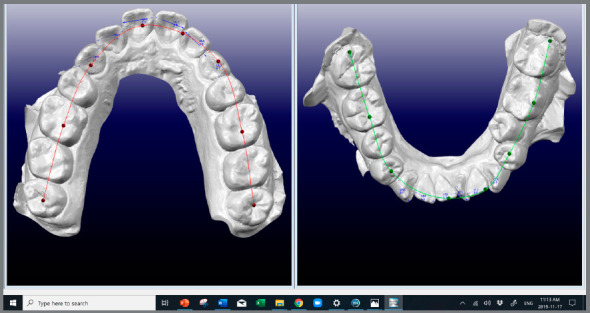
OrthoCAD™ for tooth, used for measurements.

Until the 1990s, orthodontists relied on clinical observations, 2D radiographs and unmounted study casts.^53^ ^-^ ^55^ Treatment planning relied heavily on both the experience of the operator and the underlying “treatment philosophies” of practice.^56^ ^,^ ^57^ Since 1993, it has been possible to visualize the true 3D malocclusion with CBCT and digital dental casts. These files were imported, manipulated, and measured in software such as OrthoCAD™^58^ ^-^ ^60^ and Dolphin imaging™ (Figs 5 and 6).

## USE OF DEEP LEARNING SOFTWARE FOR TREATMENT PLAN SIMULATIONS

Despite these technological advances, the diagnosis and treatment planning were still performed in the conventional way. These technologies were often reserved for complex malocclusions and orthognathic surgery, and not applied to more routine cases.^61^ ^,^ ^62^

**Figure 7:**
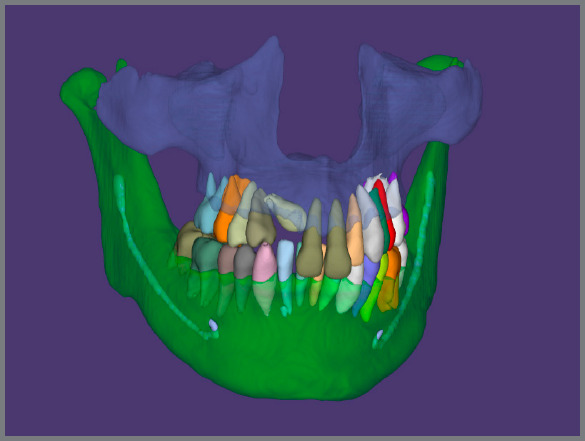
Segmentation of CBCT data using the Diagnocat™ software (Diagnocat Inc.) and deep learning algorithms.

**Figure 8:**
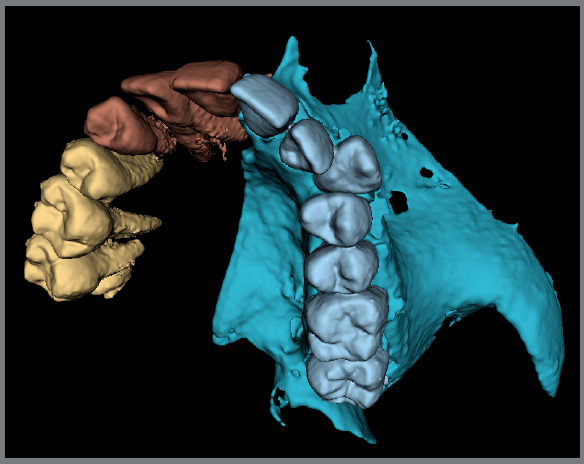
Segmented maxilla showing the bony contours and the roots.

In 1997, Invisalign™ introduced automatic segmentation of teeth, to perform virtual tooth movement and create digital treatment simulations. Recently, several companies such as CephX™ and Diagnocat™ have developed deep learning algorithms to label teeth and cephalometric points, and automatically segment DICOM data into STL. This process allowed the files to be fused with the more detailed intraoral STL files, and imported into tooth moving software.^63^ ^-^ ^67^ Another company, Cavycon™, has developed a virtual articulator using an open-source software (Blender) (Figs 7 and 8).

**Figure 9:**
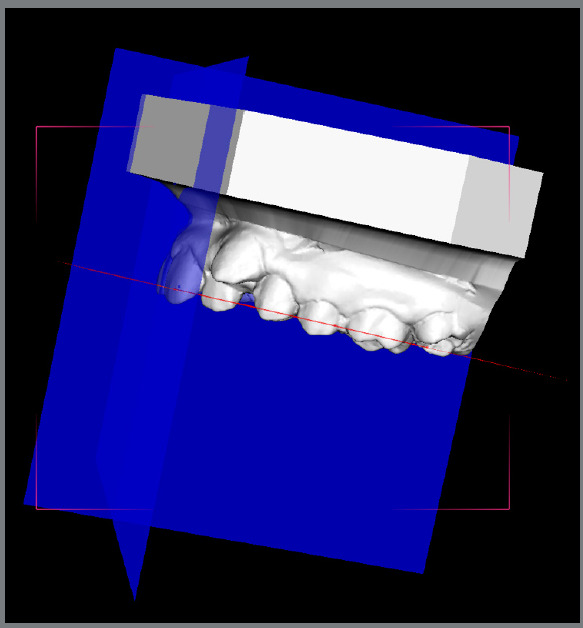
XYZ coordinates using DDP software.

**Figure 10:**
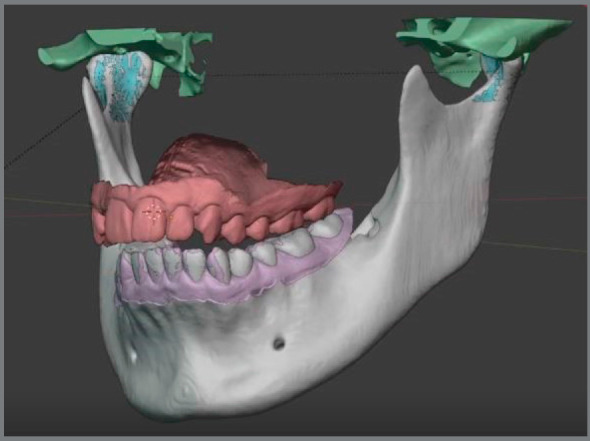
Virtual articulator based on segmented CBCT data, using Blender add-on ( Cavycon ).

These segmentation and fusion processes allow for significant improvement in orthodontic diagnosis and treatment planning. The possibility to virtually correct malocclusions in all three planes of space while incorporating the hard and soft tissues into the plan is available to the clinician with some computer knowledge. A virtual patient is created from the initially distinct datasets, which are fused, segmented and properly oriented to allow for the creation of realistic simulations^68^ ^,^ ^69^ (Figs 9 and 10).

Biologic bone and soft tissue modeling and remodeling principles must be carefully considered. At this time, it cannot be accurately calculated. Despite its fairly high learning curve and time-consuming process, this powerful virtual environment enables the clinician to incorporate deep learning concepts into fully individualized orthodontic treatment planning.^70^

## “INVERT, ALWAYS INVERT”

**Figure 11:**
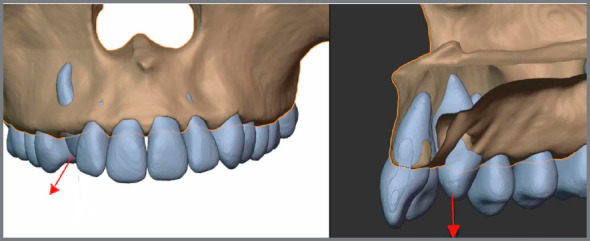
Digitally determined line of force of an impacted canine, created by a Python script in Blender software.

Once the virtual patient is created using these technologies, a deep neural network is used to predict the most appropriate treatment plan to correct the malocclusion within the physiological boundaries, and minimize treatment morbidity. The treatment simulation software and 3D occlusogram have the potential to virtually correct the malocclusion, determine the sequence or staging of treatment, and establish the biomechanical systems to be used (Fig 11). 

## WHAT CAN DEEP LEARNING AND ESPECIALLY UNSUPERVISED LEARNING TEACH US? OPTIMIZE OUR OUTCOMES AND BE MORE EFFICIENT. DATA VS METHOD

It was demonstrated that by improving the quantity and quality of data and optimizing data labelling and classification, the outcome was improved by more than 15%,^71^ far greater than the improvement from developing new methods or appliances. Data science has a tremendous potential to change the way orthodontics is performed, as practitioners tend to focus more on creating perfect tools than leveraging individual patient data.^72^ The use of neural networks presents a considerable potential to improve the predictability of our treatments.^73^ The future that is built around the age of individualization and prediction will be to move toward a more data-centric model of diagnosis and treatment planning, and away from an experiential model.

## AUGMENTED INTELLIGENCE TO THE SERVICE OF THE ORTHODONTIST?

1. Required diagnostic data:

a) Intraoral scan.
b) A centric relation registration.
c) Intra and extraoral photographs with reference points.
d) CBCT or 2D cephalograms (lateral and PA) oriented from the facial photographs.

In this section, neural networks may be used to automatically recognize points, perform measurements and segment intraoral scans and CBCT data. The use of these networks will speed up and enhance the diagnostic capabilities of the orthodontist. 

2. Data analysis software

The data is then entered in a neural network where simulations are run using different parameters and algorithms. The case is then virtually finished to the highest standards, using the machine learning algorithms and the experience of the orthodontist^51^. One or several treatment options may be developed, evaluated, and appraised at that time (e.g., extraction vs non-extraction, surgical vs non-surgical). Once the orthodontist decides on the most appropriate treatment outcome, the staging is set; biomechanics, determined; and appliances, individually fabricated using 3D printed models and templates. 

## IS AI A BENEFIT OR A CURSE FOR OUR PROFESSION?

AI is changing our profession mainly without our involvement. Once shared with third parties, the clinician no longer owns or control the data. The companies manipulate the data, return a finished product without sharing the process. This is a feedforward process. As previously mentioned, data mining has the potential to unleash new treatment management that may be offered to any dental practitioners. Companies do offer their products to the widest possible market, and it is the responsibility of our profession to develop, maintain and protect our own algorithms into the hands of skillfully trained orthodontists. One method would be to develop a data repository (Data Management Coordination Center) and have our professional organizations create development platforms for research and development that result in product development that will benefit our profession. 

Artificial Intelligence in dentistry is used mainly as narrow intelligence, which means that computers can accomplish one task at a time very efficiently if enough data is available and if training is provided by the programmer. Computers do not possess human intelligence and have no moral compass, no framework of compassion, intuition, or emotions. The state of consciousness, as defined by Kant in his book “Critique of Pure Reason”,^74^ is totally absent in computers, which makes them totally unaware of their environment. AI is therefore very far and very different from human intelligence. 

The most promising avenue for the profession is to continue to develop augmented intelligence where the power of AI is harnessed by the orthodontist to the benefit of the patient. Orthodontist will realize that they possess and control the data necessary to optimize augmented intelligence. Technology is not a curse, but an opportunity to improve our profession, and it must be adopted within very strict boundaries. 

## CONCLUSIONS

Artificial Intelligence and Deep Learning are evolving at a galloping pace. The potential to harness their capacity to analyze large datasets and develop realistic treatment predictions is a promising advancement for Orthodontics. Experience-based diagnosis and treatment planning have greatly benefited the profession. The profession is now moving rapidly towards Orthodontic 4.0: “The age of treatment prediction, individualization and simulation”. The advent of augmented intelligence based on algorithms mining large datasets has the potential to expand the horizons of the orthodontist to improve the quality of life of our future orthodontic patients if controls on data mining are established with third parties. 
